# Robotic‐arm assisted total knee arthroplasty has a learning curve of 16 cases and increased operative time of 12 min

**DOI:** 10.1111/ans.17975

**Published:** 2022-08-12

**Authors:** Mei Lin Tay, Matthew Carter, Nina Zeng, Matthew L. Walker, Simon W. Young

**Affiliations:** ^1^ Department of Surgery, Faculty of Medical and Health Sciences (FMHS) University of Auckland Auckland New Zealand; ^2^ Department of Orthopaedic Surgery North Shore Hospital, Waitematā DHB Auckland New Zealand; ^3^ Stryker New Zealand Auckland New Zealand

**Keywords:** knee replacement, learning curve, operative time, robot, total knee arthroplasty

## Abstract

**Background:**

Robotic‐arm assisted systems are increasingly used for knee arthroplasty, however introduction of new systems can involve a learning curve. We aimed to define the learning curve in terms of operative time and component placement/sizing of a robotic system for total knee arthroplasty (TKA) in a team of experienced surgeons, and to investigate mid‐term patient outcomes.

**Methods:**

A total of 101 consecutive patients underwent primary robotic‐arm assisted TKA by three surgeons (mean 2 year follow‐up). Operative times, component placement, implant sizing and reoperations were recorded. Cumulative Summation (CUSUM) was used to analyse learning curves. Patient outcomes were compared between learning and proficiency phases.

**Results:**

The learning curve was 16 cases, with a 12‐min increase in operative time (*P* < 0.01). Once proficiency was achieved, the greatest time reductions were seen for navigation registration (*P* = 0.003) and bone preparation (*P* < 0.0001). A learning curve was found with polyethylene (PE) insert sizing (*P* = 0.01). No differences were found between learning and proficiency groups in terms of implant survival (100% and 97%, respectively, NS) or patient‐reported outcome measures at 2 years (NS).

**Conclusion:**

Introduction of a robotic‐arm assisted system for TKA led to increased operative times for navigation registration and bone preparation, and a learning curve with PE insert sizing. No difference in patient outcomes between learning and proficiency groups at 2 years was found. These findings can inform surgeons' expectations when starting to use robotic‐assisted systems.

## Graphical abstract

The learning curve for a robotic‐arm assisted system for total knee arthroplasty (TKA) was 16 cases and this led to increased operative time of 12 min. There was also a learning curve for polyethylene insert sizing, but not the other component placement or sizing. At 2 year follow‐up there was no difference in implant survival, reoperations or patient reported outcomes.

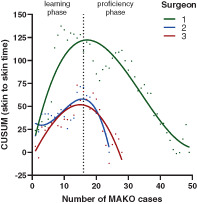

## Introduction

Total knee arthroplasty (TKA) is an effective treatment for patients with end‐stage osteoarthritis. Early aseptic TKA failures, mainly attributed to aseptic loosening,^1^ may be avoided with improved surgical accuracy.^2^ Robotic‐arm assisted systems were introduced to increase precision in implant positioning and balancing.^3^, ^4^ However, new surgical techniques can involve learning curves, typically associated with longer operative times and increased risk of adverse events.^5^, ^6^

Learning can vary among surgeons,^7^ and is linked to experience and volume.^8^, ^9^ Further reporting of learning curves, representing the rate of learning a new task over time, associated with such systems will allow for generalisability to a broader range of surgeons. Additionally, while robotic‐assisted systems are increasingly used,^10^, ^11^ to date there is limited information on the impact of a learning curve on patient outcomes past early follow‐up of 1–2 months.^12^

Therefore, the aims of this study were to: (1) report the learning curves in terms of operative time and component placement/sizing of robotic‐assisted TKA in a surgical team of experienced consultants, and (2) investigate the impact of learning curves on longer‐term patient outcomes. The hypothesis was that there would be learning curves in terms of operative times, component sizing and patient outcomes.

## Patients and methods

Ethical approval for this study was obtained from national and local committees. This study included prospectively‐collected data from surgeons who completed at least 20 robotic‐arm assisted TKA procedures in a public hospital setting between February 2018 and November 2020. The surgeons were senior arthroplasty consultants performing robotic‐assisted TKA surgery for the first time, however all were experienced with computer‐navigated TKA. There were 101 consecutive patients undergoing primary TKA for osteoarthritis.

### Surgical technique

The MAKO Robotic Interactive Orthopaedic (RIO) system (Stryker, Kalamazoo, MI) was used. A medial parapatellar incision was performed and bi‐cortical navigation pins inserted in the femur and tibia for intra‐operative navigation. Registration of landmarks was performed according to the Mako Surgical protocol.^13^ Medial and lateral osteophytes were removed and intraoperative limb alignment was measured. Virtual implant gaps were manually captured in extension (0°–15°) and in 90° of limb flexion. Component position, sizing and insert thickness were virtually adjusted to balance gaps to around 20 mm, with soft tissue release as required. An integrated Mako saw handpiece (Stryker) was used for femoral and tibial resections, and a freehand saw was used for patella resurfacing. After surgeon manual assessment with trial components, the final polyethylene (PE) and definitive components were implanted. The implants were cemented Triathlon CR femur with CS polyethene prostheses (Stryker).

### Outcome measures

#### Operative time

Total operative time was recorded from incision to dressing application. The procedure was categorized into: (1) surgical approach/bone registration, (2) joint balancing, (3) bone preparation, (4) implant trialling, (5) cement implantation, and (6) closure.

#### Patient outcomes

Clinical chart review was performed in August 2021 to identify reoperations. Revision was defined as the addition or exchange of one or more component(s) or confirmed periprosthetic joint infection. Patient age, gender, body mass index (BMI) and American Society of Anesthesiologists (ASA) status were recorded. The patient cohort were 39% male, with mean age of 68.2 ± 9.6 (standard deviation) and mean BMI of 29.6 ± 4.6 (Table S1). Patient‐reported outcomes measures (PROMs) were recorded with the Oxford Knee Score (OKS),^14^ EuroQol‐5D (EQ‐5D‐5L)^15^ and Forgotten Joint Score (FJS‐12).^16^ The OKS and EQ‐5D‐5L were administered pre‐operatively and then post‐operatively with FJS‐12 at 6 weeks, 1 year, and 2 years.

#### Differences between pre‐surgical and final component plans

Component position and size from the virtual initial pre‐operative and final intra‐operative plans were recorded in the coronal, sagittal and axial planes, as previously described.^17^, ^18^ Briefly, the femoral mechanical axis was defined as the center of the femoral head to the center of the distal femur, while the tibial mechanical axis was defined as the center of the proximal tibia to the center of the ankle, defined as a lateral: medial ratio of 56:44 between malleoli. Femoral rotation was the angle between the surgical trans‐epicondylar axis and the posterior condylar axis of the implant. Tibial rotation was calculated between the anteroposterior axis of the tibial implant and Akagi's Line.^19^ Resection heights were measured virtually between resection landmarks and the closest point on planned resections. The surgical plans were based on a target insert size of 9 mm for the majority of cases (*n* = 98) and 11 mm (three cases). Planned and final PE insert sizes were recorded.

### Statistical analysis

A Cumulative Summation (CUSUM) method^9^, ^20^ was used to analyse learning curves for operative time. Mean operative times and component positioning measurements were calculated for every 10 consecutive cases performed.^4^ Using the minimally clinically important difference (MCID) for OKS of 5^21^ and standard deviation of 7.2,^22^ 33 participants were needed per group to detect differences in PROMs (80% power, *α* = 0.05). Between‐group differences were calculated using Fisher's or Chi‐squared tests for categorical data, *t*‐tests or 1‐way ANOVA for normally‐distributed continuous variables, and Mann–Whitney or Kruskal‐Wallis tests for non‐parametric variables. Revision and non‐revision reoperation curves were analysed using Kaplan–Meier and log‐rank (Mantel‐Cox) tests. A *P*‐value <0.05 was considered significant. Statistical analyses were performed with IBM SPSS Statistics v26 (IBM corp., Armonk, NY, USA) and PRISM 8 (GraphPad, San Diego, CA).

## Results

During the study period, three surgeons each completed between 24 and 49 robotic‐assisted TKAs (Table S2).

### Operative times

**Fig. 1:**
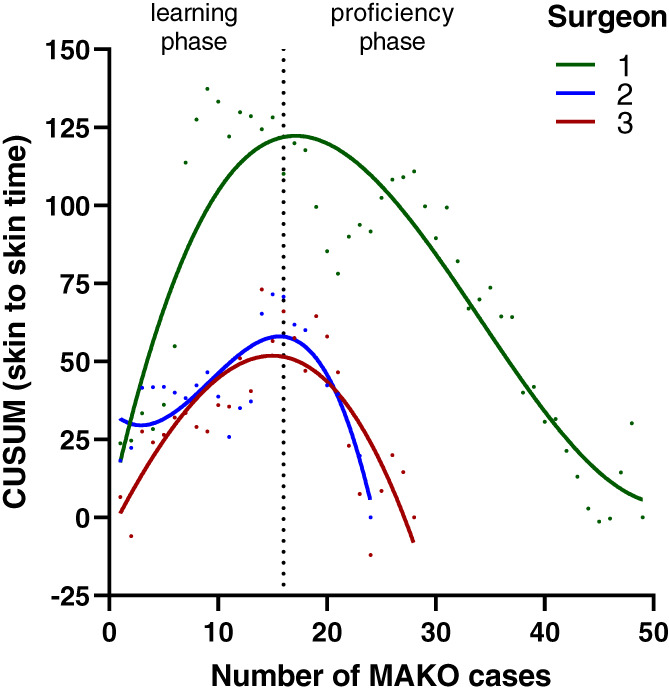
CUSUM (cumulative summation) analysis of learning curves (operative time) of robotic‐arm assisted TKA for three experienced surgeons. The mean inflexion point of 16 cases is represented by a vertical dotted line and represents the change from learning phase to proficiency.

The inflexion point was at the 16th case (*r* ^2^ 0.64–0.83; Fig. 1), which distinguished between learning and proficiency phases. There were no differences in baseline patient demographics for the two groups (NS, Table S3). Total operative time in the learning phase was 12 min longer than in the proficiency phase (90.3 cf. 78.4 min; *P* < 0.0001). This was attributed to increased time in the surgical approach and bone registration (up to 4 min longer, *P* = 0.003), and bone preparation (up to 5 min longer, *P* < 0.0001; Table 1), and limited to the first 10 cases performed by each surgeon (*P* < 0.05; Table S4).

**Table 1:** Operative times of the surgical stages in robotic‐arm assisted TKA, per 10 surgeon cases

Surgical stage (mins)	Cases 1–10 (*n* = 30)	Cases 11–20 (*n* = 30)	Cases 21–30 (*n* = 22)	Cases 31–40 (*n* = 10)	Cases 41–50 (*n* = 9)	*P*‐value
Approach + bone registration	15.9 ± 5.3 (9–34)	13.3 ± 3.3 (7–20)	11.5 ± 2.8 (8–17)	12.9 ± 1.6 (11–16)	15.1 ± 2.4 (11–18)	0.003*
Joint balancing	8.4 ± 4.3 (3–21)	8.0 ± 3.7 (2–17)	7.1 ± 3.4 (2–16)	6.7 ± 1.7 (4–9)	5.7 ± 2.1 (3–9)	0.42
Bone preparation	13.0 ± 6.0 (6–26)	8.8 ± 3.7 (1–23)	8.5 ± 5.0 (2–25)	8.2 ± 4.8 (5–21)	8.2 ± 5.8 (5–23)	<0.0001*
Component trialling	18.7 ± 7.8 (6–47)	18.6 ± 7.7 (7–38)	17.8 ± 4.5 (12–28)	17.1 ± 6.4 (8–29)	20.4 ± 4.1 (17–27)	0.86
Cement Implantation	11.3 ± 3.7 (3–16)	12.5 ± 5.0 (4–21)	9.2 ± 5.2 (3–17)	9.8 ± 4.9 (3–15)	12.8 ± 2.3 (9–15)	0.20
Closure	21.8 ± 3.3 (16–28)	20.5 ± 5.7 (12–35)	21.7 ± 6.1 (15–38)	18.4 ± 5.3 (11–26)	19.8 ± 3.7 (14–25)	0.27
Total operative time	92.1 ± 14.2 (69–140)	84.4 ± 14.3 (63–121)	79.0 ± 11.4 (58–102)	75.3 ± 11.0 (57–91)	77.8 ± 14.0 (51–97)	0.003*

*Note*: Operative time in minutes presented as mean and standard deviation, with ranges presented within brackets.

* Significant between group differences (*P* < 0.05).

### Pre‐surgical *vs.* final component plans

**Fig. 2:**
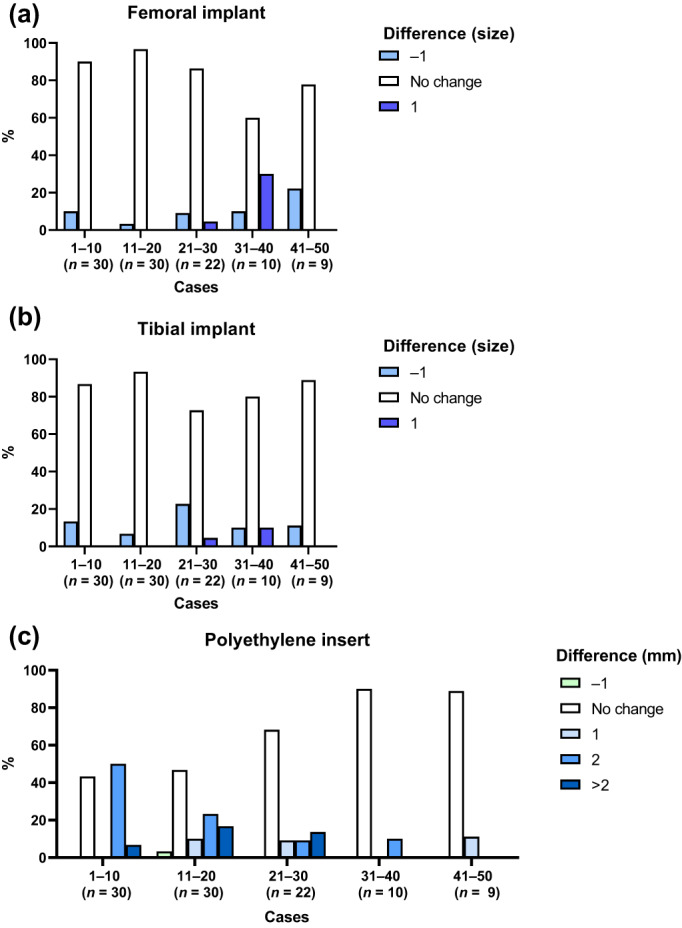
Proportion of change in component sizing (pre‐ *vs*. post‐plan) during introduction of robotic‐arm assisted TKA, per 10 surgeon cases, for (a) femoral component (NS across groups), (b) tibial implant (NS), and (c) polyethylene insert (*p* = 0.02).

There was no evidence of a learning curve with component planning, despite some between‐group differences with posterior lateral resection (*P* = 0.004), proximal medial resection (*P* = 0.03) and proximal lateral resection (*P* = 0.03; Table S5). There was a learning curve for PE insert sizing: accuracy of improved from 43% in the first 10 cases to 89% in the last 10 cases (*P* = 0.02, Fig. 2).

### Patient outcomes

Revision‐free implant survival at mean follow‐up of 2 years was 100% and 97% for the learning and proficiency phases, respectively (NS, Fig. S1a). Non‐revision reoperation‐free survival at 2 years was 98% and 95% for the learning and proficiency phases, respectively (NS, Fig. S1b). There was one revision: a liner exchange, and four other reoperations: one patient from the learning phase (2%) underwent manipulation under anaesthetic, and three patients from the proficiency phase (6%) underwent washouts (Tables S6–7).

At 2‐years, the PROM response rate was 56% and 67% for the learning and proficiency groups, respectively. Patients operated on during the proficiency phase reported higher scores for the EQ‐5D‐5L index score at 1 year (0.9 cf. 0.8, *P* = 0.0004), the EQ‐5D‐5L VAS at 2 years (89 cf. 74, *P* = 0.01), and for the FJS‐12 at 6 weeks (57 cf. 37, *P* = 0.01) and 1 year (70 cf. 55, *P* = 0.02; Fig. S2, Table S8).

## Discussion

The main finding was robotic‐assisted TKA had a learning curve in terms of operative time of 16 cases in an experienced surgical team with medium to high volumes. Using the same robotic system, Kayani *et al*. reported a learning curve of seven cases for a single high‐volume surgeon,^4^ and Vermue *et al*. reported learning curves between 11 and 43 for three high‐volume surgeons.^9^ In our study the number of cases needed to achieve proficiency was 16, which was higher than the 11 cases previously reported for unicompartmental knee arthroplasty (UKA) using the system.^18^ These findings can be used in conjunction with previous reports to inform surgeon expectations of learning curves of robotic systems. An increase of 12 min in operative time found during the learning phase was attributed to the registration and bone preparation stages, in agreement with previous studies.^4^, ^18^

Previous studies reported that the learning curves did not lead to more early complications.^4^, ^9^ We found no further risk of complications in the mid‐term (2 years). While more patients from the proficiency phase group required reoperation, two of the three non‐revision reoperations occurred from trauma not directly related to the surgical procedure. No difference in 2‐year implant survivorship for the learning (100%) and proficiency (97%) phases was found; these were comparable with registry reports (98% at 2 years).^10^

A learning curve was found with sizing of the PE insert, but not with tibial or femoral component sizing, consistent with previous findings with the UKA system.^18^ Optimal component sizing can influence TKA implant longevity.^23^ Thinner PE inserts (6–8 mm) may lead to higher contact stresses and increased wear,^24^, ^25^, ^26^ associated with release of particulate debris that can ultimately lead to osteolysis and aseptic loosening.^27^, ^28^ Conversely, thicker PE inserts (>11–16 mm) are also linked to lower implant survival. These may be associated with higher strain on tibial bone leading to overload and collapse of cancellous bone.^29^, ^30^ In this study, there was evidence of a learning curve with PE sizing, with larger than planned insert sizes implanted during the learning phase. This suggests that the difficulties in achieving optimal PE thickness may reflect a learning curve in achieving ligament balance with the robotic system, and therefore identified an area where extra care may be warranted during the learning phase.

Clinical importance of PROM differences found between learning and proficiency groups was unclear. A previous study reported no differences in International Knee Society Score (IKSS) and Short Form 12 (SF‐12) scores at 1 year.^7^ In our study, patients in the learning phase group had lower EQ‐5D VAS scores but no clinically meaningful difference in the index score at 2 years,^31^ suggesting this was related to non‐knee related health issues. While the FJS‐12 is robust, its administration as a post‐operative metric^16^ may be affected by baseline differences. The score was lower during the learning phase, however change scores were similar: 18 and 13 at 1 year, and 27 and 23 at 2 years for the learning and proficiency phases, respectively. The FJS‐12 scores after surgeon proficiency were high (70 at 1 year; 80 at 2 years) compared with other studies (38 and 59 at 1 year for robotic‐assisted or navigated TKA^32^, ^33^; between 42 and 46 in manual TKA^5^, ^32^). Combined with a lack of between‐group differences in the ‘gold standard’ OKS, it could suggest that patient outcomes achieved during the learning phase were comparable to outcomes achieved with manual TKA, but once surgeons achieved proficiency they could achieve more desirable outcomes.

This study had several limitations. First, as above, we reported a novel difference in FJS‐12 scores between the learning and proficiency groups, however we did not have baseline scores. Despite this, comparisons could be made with outcomes from other TKA groups. PROM response rates were relatively low at times, however 60% response is typical for PROMs without active follow‐up, and was not expected to introduce significant sample bias.^34^ Second, long‐leg radiographs were unavailable for verification of post‐surgical component placements. Other studies have validated the accuracy of the CT‐based measurements recorded by the MAKO system,^3^, ^20^ providing a degree of confidence in these measurements. Third, the surgeons included in this study were senior arthroplasty consultants with experience in computer navigated TKA. Therefore, the findings of this study may hold more relevance for surgeons with similar experience in computer navigation who are moving to robotic‐assisted procedures. Finally, the findings presented here were limited to mid‐term follow‐up as the use of the robotic‐arm assisted system for TKA began in 2018. We plan to continue monitoring patients longer‐term.

In summary, introduction of a robotic‐arm assisted system for TKA in an experienced surgical team had a learning curve of 16 cases for increased operative time in the navigation registration and bone preparation phases, and for PE insert sizing. No difference in patient outcomes between learning and proficiency groups at 2 years was found. These findings have identified areas that may require extra care with introduction of robotic‐assisted systems for knee arthroplasty.

## Author contributions

**Mei Lin Tay:** Conceptualization; data curation; formal analysis; investigation; methodology; validation; visualization; writing – original draft; writing – review and editing. **Matthew Carter:** Conceptualization; data curation; formal analysis; methodology; validation; writing – review and editing. **Nina Zeng:** Data curation; validation; writing – review and editing. **Matthew L. Walker:** Investigation; methodology; writing – review and editing. **Simon W. Young:** Conceptualization; investigation; methodology; supervision; writing – review and editing.

## Conflict of interest

SWY receives research support and is a paid consultant for Stryker Orthopaedics NZ. MC is a senior product specialist at Stryker Orthopaedics. MLW is a paid consultant for Stryker Orthopaedics. These affiliations did not influence study design or data collection, analysis or interpretation of data. Stryker Orthopaedics did not influence any decisions for manuscript preparation or publication. MLT and NZ declare no conflict of interest. This research did not receive any specific grant from funding agencies in the public, commercial, or not‐for‐profit sectors.

## Supporting information


**Figure S1**. Kaplan–Meier survival curves comparing rates of (a) revisions and (b) non‐revision reoperations for the learning and proficiency phases of robotic‐arm assisted TKA. There were no significant differences (*P* = 0.25 and *P* = 0.08, respectively) between curves.
**Figure S2**. Patient‐reported outcome measures (PROM) of robotic‐arm assisted TKA. Cases performed during the learning phase are represented by dotted lines and cases performed during the proficiency phase are represented by solid lines. *OKS*, Oxford Knee Score; *EQ‐5D‐5L*, EuroQol 5D; *FJS‐12*, Forgotten Joint Score. *significant differences between learning and proficiency groups (*P* < 0.05).
**Table S1**. Baseline demographics of patients undergoing robotic‐arm assisted TKA
**Table S2**. Surgeon characteristics
**Table S3**. Comparison of patient characteristics and surgical details between the learning curve phases with robotic‐arm assisted TKA
**Table S4**. Increased operative times of robotic‐arm assisted TKA during the learning phase, per 5 surgeon cases
**Table S5**. Differences in implant positioning of pre‐surgical plan compared with final implant plan, per 10 cases, during introduction of robotic‐arm assisted TKA
**Table S6**. Revision details of patients undergoing robotic‐assisted total knee arthroplasty
**Table S7**. Non‐revision reoperation details of patients undergoing robotic‐assisted total knee arthroplasty
**Table S8**. Comparison of patient reported outcome measures in learning and proficiency phases of robotic‐assisted total knee arthroplasty
