# Supplementary material for: Robotic‐arm assisted total knee arthroplasty has a learning curve of 16 cases and increased operative time of 12 min
Source: ANZ J Surg. 2022 Aug 12;92(11):2974–9. doi: 10.1111/ans.17975 (PMC9804534; doi:10.1111/ans.17975)
Supplement: Supplementary file 1 — Figure S1. Kaplan–Meier survival curves comparing rates of (a) revisions and (b) non‐revision reoperations for the learning and proficiency phases of robotic‐arm assisted TKA. There were no significant differences (P = 0.25 and P = 0.08, respectively) between curves. Figure S2. Patient‐reported outcome measures (PROM) of robotic‐arm assisted TKA. Cases performed during the learning phase are represented by dotted lines and cases performed during the proficiency phase are represented by solid lines. OKS, Oxford Knee Score; EQ‐5D‐5L, EuroQol 5D; FJS‐12, Forgotten Joint Score. *significant differences between learning and proficiency groups (P < 0.05). Table S1. Baseline demographics of patients undergoing robotic‐arm assisted TKA Table S2. Surgeon characteristics Table S3. Comparison of patient characteristics and surgical details between the learning curve phases with robotic‐arm assisted TKA Table S4. Increased operative times of robotic‐arm assisted TKA during the learning phase, per 5 surgeon cases Table S5. Differences in implant positioning of pre‐surgical plan compared with final implant plan, per 10 cases, during introduction of robotic‐arm assisted TKA Table S6. Revision details of patients undergoing robotic‐assisted total knee arthroplasty Table S7. Non‐revision reoperation details of patients undergoing robotic‐assisted total knee arthroplasty Table S8. Comparison of patient reported outcome measures in learning and proficiency phases of robotic‐assisted total knee arthroplasty [file ANS-92-2974-s001.docx]

**Supporting information**


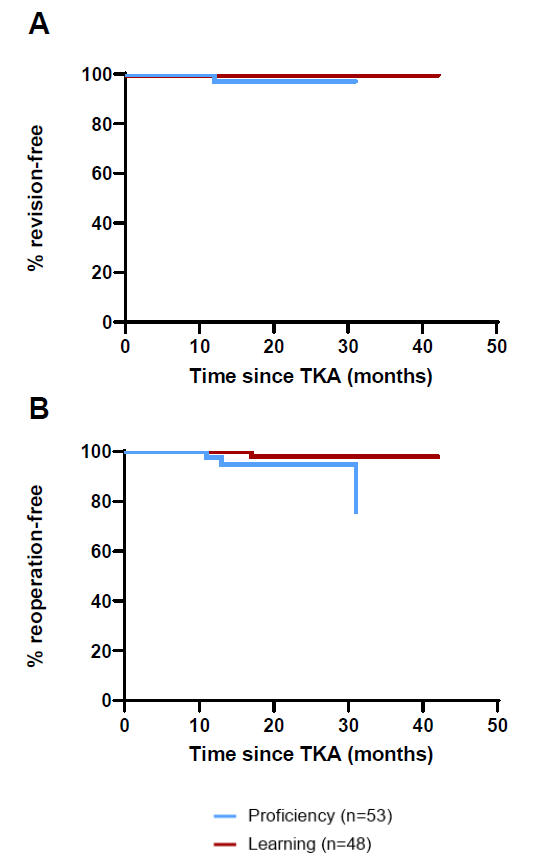


**Fig. S1** Kaplan-Meier survival curves comparing rates of A) revisions and B) non-revision reoperations for the learning and proficiency phases of robotic-arm assisted TKA. There were no significant differences (p=0.25 and p=0.08, respectively) between curves.


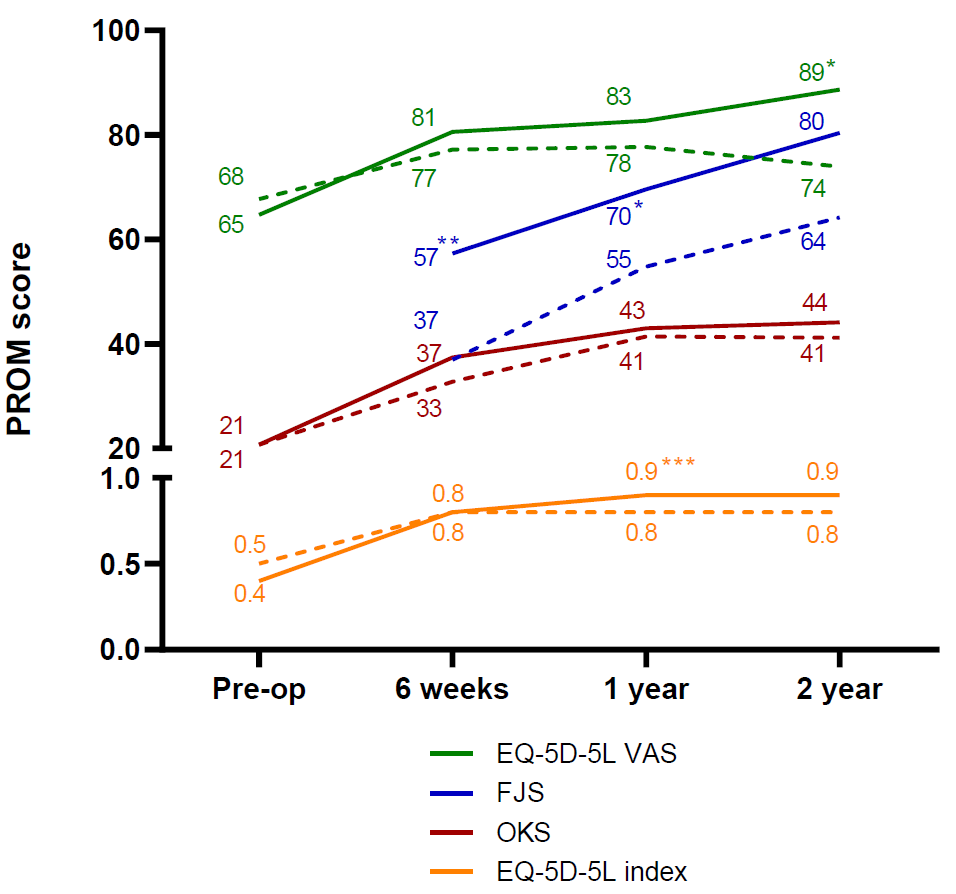


**Fig. S2** Patient-reported outcome measures (PROM) of robotic-arm assisted TKA. Cases performed during the learning phase are represented by dotted lines and cases performed during the proficiency phase are represented by solid lines. *OKS*, Oxford Knee Score; *EQ-5D-5L*, EuroQol 5D; *FJS-12*, Forgotten Joint Score. *significant differences between learning and proficiency groups (p<0.05).

**Table S1** Baseline demographics of patients undergoing robotic-arm assisted TKA

| **Patient population (n)** |  |
| --- | --- |
| **Total** |  |
| Knees | 101 |
| Patients | 92 |
| **Gender** |  |
| Male | 39 (39%) |
| Female | 62 (61%) |
| **Age at surgery (years)** |  |
| Mean ± std dev | 68.2 ± 9.6 |
| Range | 50-88 |
| **BMI (kg/m^2^)** |  |
| Mean ± std dev | 29.6 ± 4.6 |
| Range | 18.5-41.0 |
| **ASA** |  |
| 1 | 10 (10%) |
| 2 | 68 (67%) |
| 3 | 23 (23%) |
| **Side of intervention** |  |
| Left | 47 (47%) |
| Right | 54 (54%) |
| **Length of stay (days)** |  |
| Mean ± std dev | 3.8 ± 1.7 |
| Range | 2-11 |
| **Length of followup (months)** |  |
| Mean ± std dev | 2.0 ± 0.8 |
| Range | 9 months - 3.5 years |

Data presented as mean and standard deviation. *ASA*, American Society of Anesthesiologists; *BMI*, body mass index; *std dev*, standard deviation.

**Table S2** Surgeon characteristics

| **Surgeon** | **Years in Practice** | **Case volume** | **Number of MAKO cases** |
| --- | --- | --- | --- |
| 1 | 16 | High | 49 |
| 2 | 7 | Medium | 28 |
| 3 | 7 | High | 24 |

Case volume was categorised to medium (50-100 cases per year) or high (>100 cases per year).

**Table S3** Comparison of patient characteristics and surgical details between the learning curve phases with robotic-arm assisted TKA

| **Patient characteristic, n(%)** | **Learning phase (n=48)** | **Proficiency phase (n=53)** | **p-value** |
| --- | --- | --- | --- |
| Male gender | 20 (42%) | 19 (36%) | 0.79 |
| Age (years) | 69.7 ± 10.4 | 67.0 ± 8.8 | 0.13 |
| BMI (kg/m^2^) | 30.2 ± 4.7 | 29.1 ± 4.4 | 0.21 |
| ASA 3 | 11 (23%) | 12 (23%) | >0.99 |
| Right side | 28 (58%) | 26 (49%) | 0.46 |
| Length of stay >4 days | 13 (27%) | 8 (15%) | 0.22 |
| Total operative time | 90.3 ± 14.9 | 78.4 ± 11.4 | <0.0001* |
| Planned vs actual PE size difference | 1.2 ± 1.3 | 0.6 ± 1.0 | 0.01* |

Data presented as mean and standard deviation. *ASA*, American Society of Anesthesiologists; *BMI*, body mass index; *PE*, polyethylene. *Significant between group differences (p<0.05).

**Table S4** Increased operative times of robotic-arm assisted TKA during the learning phase, per 5 surgeon cases

| **Surgical stage (mins)** | **Cases 1 - 5 (n=15)** | **Cases 6 - 10 (n=15)** | **Cases 11-16 (n=18)** |
| --- | --- | --- | --- |
| Increased operative time | 13.7 ± 13.8 (-9.4-36.6) | 13.7 ± 15.1 (-1.4-61.6) | 8.9 ± 15.9 (-15.4-42.6) |

Measurements are presented as mean and standard deviation, with ranges presented within brackets.

**Table S5** Differences in implant positioning of pre-surgical plan compared with final implant plan, per 10 cases, during introduction of robotic-arm assisted TKA

| **Component positioning (mm)** | **Cases 1 - 10 (n=30)** | **Cases 11 - 20 (n=30)** | **Cases 21 - 30 (n=22)** | **Cases 31 - 40 (n=10)** | **Cases 41 - 50 (n=9)** | **p-value** |
| --- | --- | --- | --- | --- | --- | --- |
| Femoral flexion (deg) | 0.1±0.9  (-2.5-3.0) | -0.2±0.8  (-2.7-2.0) | -1.2±2.3  (-6-3.3) | -0.2±2.3  (-4-4.1) | -0.3±1.4  (-2.5-2.4) | 0.14 |
| Distal medial resection | -0.3±1.1  (-3.5-2.0) | -0.5±0.9  (-3.5-1.0) | -0.6±1.5  (-3.0-3.5) | 0.3±1.3  (-0.5-3.5) | -0.3±0.4  (-1.0-0) | 0.61 |
| Distal lateral resection | -0.6±1.0  (-2.5-2.0) | -0.6±1.1  (-3.5-2.5) | -0.8±1.8  (-4.5-3.0) | -0.3±0.7  (-1.5-0.5) | -0.4±0.8  (-2.0-0.5) | 0.78 |
| Posterior medial resection | 1.2±1.0  (0-3.5) | 1.2±1.5 (-4.0-3.5) | 1.7±1.5  (-1.0-5.5) | 0.5±1.7 (-1.5-3.5) | 1.1±1.2  (0-3.5) | 0.16 |
| Posterior lateral resection | 0.3±1.1  (-2.0-2.5) | 0.3±1.0  (-1.5-2.5) | 1.1±1.8  (-3.5-4.5) | -0.8±1.5  (-3.0-1.5) | 1.1±0.9  (0-2.5) | 0.004* |
| Tibial slope (deg) | 0.2±0.6  (0-2.5) | 0±0.2  (-1/0-0) | 0±0.3  (-1.0-1.0) | 0±0  (0-0) | 0±0  (0-0) | 0.13 |
| Proximal medial resection | 0.7±1.4  (-2.0-4.0) | -0.1±1.8  (-3.5-4.0) | -0.5±1.3  (-4.5-1.5) | -0.6±2.0  (-2.5-3.0) | -0.4.±1.2  (-2.5-1.5) | 0.03* |
| Proximal lateral resection | -0.1±1.1  (-3.5-2.0) | -0.7±1.3  (-4.0-2.5) | -1.1±1.2  (-3.0-0.5) | -0.9±1.7  (-3.0-2.0) | -1.2±1.3  (-2.5-4.0) | 0.03* |

Measurements are presented as mean and standard deviation, with ranges presented within brackets. *Significant between group differences (p<0.05).

**Table S6** Revision details of patients undergoing robotic-assisted total knee arthroplasty

| **Patient** | **Time post-surgery** | **Revision details** | **Reason for revision** | **Phase** |
| --- | --- | --- | --- | --- |
| 109 | 7 months | Liner exchange | Instability | Proficiency |

**Table S7** Non-revision reoperation details of patients undergoing robotic-assisted total knee arthroplasty

| **Patient** | **Time post-surgery** | **Reoperation details** | **Reason for reoperation** | **Phase** |
| --- | --- | --- | --- | --- |
| 32 | 1 month | Washout | Wound dehisence (trauma) | Proficiency |
| 93 | 2 months | MUA | Stiffness | Learning |
| 118 | 1 month | Washout | Haemarthroses | Proficiency |
| 102 | 2 months | Washout | Wound dehisence (trauma) | Proficiency |

*MUA*, manipulation under anaesthesia.

**Table S8** Comparison of patient reported outcome measures in learning and proficiency phases of robotic-assisted total knee arthroplasty

| **PROMs** | **Learning phase (n=48)** | **Proficiency phase (n=53)** | **p-value** |
| --- | --- | --- | --- |
| ***Pre-op*** | ***71% response*** | ***74% response*** |  |
| OKS | 21 ± 7 | 21 ± 8 | >0.99 |
| EQ-5D-5L Index Score | 0.5 ± 0.2 | 0.4 ± 0.3 | 0.10 |
| EQ-5D-5L VAS | 68 ± 18 | 65 ± 19 | 0.50 |
| ***6 weeks*** | ***60% response*** | ***42% response*** |  |
| OKS | 33 ± 10 | 37 ± 9 | 0.10 |
| EQ-5D-5L Index Score | 0.8 ± 0.1 | 0.8 ± 0.1 | >0.99 |
| EQ-5D-5L VAS | 77 ± 14 | 81 ± 13 | 0.38 |
| FJS-12 | 37 ± 26 | 57 ± 31 | 0.01* |
| ***1 year*** | ***58% response*** | ***83% response*** |  |
| OKS | 41 ± 6 | 43 ± 5 | 0.28 |
| EQ-5D-5L Index Score | 0.8 ± 0.1 | 0.9 ± 0.1 | 0.0004* |
| EQ-5D-5L VAS | 78 ± 16 | 83 ± 12 | 0.18 |
| FJS-12 | 55 ± 25 | 70 ± 24 | 0.02* |
| ***2 year*** | ***56% response*** | ***67% response*** |  |
| OKS | 41 ± 8 | 44 ± 4 | 0.23 |
| EQ-5D-5L Index Score | 0.8 ± 0.2 | 0.9 ± 0.1 | 0.12 |
| EQ-5D-5L VAS | 74 ± 18 | 89 ± 8 | 0.01* |
| FJS-12 | 64 ± 30 | 80 ± 16 | 0.09 |

Data presented as mean and standard deviation. *OKS*, Oxford Knee Score; *EQ-5D-5L*, EuroQol 5D; *FJS-12*, Forgotten Joint Score; *significant differences, learning vs. proficiency phases (p<0.05).
